# The significant other: splicing by the minor spliceosome

**DOI:** 10.1002/wrna.1141

**Published:** 2012-10-16

**Authors:** Janne J Turunen, Elina H Niemelä, Bhupendra Verma, Mikko J Frilander

**Affiliations:** Institute of Biotechnology, University of HelsinkiHelsinki, Finland

## Abstract

The removal of non-coding sequences, introns, from the mRNA precursors is an essential step in eukaryotic gene expression. U12-type introns are a minor subgroup of introns, distinct from the major or U2-type introns. U12-type introns are present in most eukaryotes but only account for less than 0.5% of all introns in any given genome. They are processed by a specific U12-dependent spliceosome, which is similar to, but distinct from, the major spliceosome. U12-type introns are spliced somewhat less efficiently than the major introns, and it is believed that this limits the expression of the genes containing such introns. Recent findings on the role of U12-dependent splicing in development and human disease have shown that it can also affect multiple cellular processes not directly related to the functions of the host genes of U12-type introns. At the same time, advances in understanding the regulation and phylogenetic distribution of the minor spliceosome are starting to shed light on how the U12-type introns and the minor spliceosome may have evolved. © 2012 John Wiley & Sons, Ltd.

## INTRODUCTION

U12-type introns were initially described as a handful of unusual introns containing non-consensus AT–AC termini and a high degree of conservation at the 5′ splice site (5′ss).^1^ These characteristics set them apart from most other introns that typically had GT–AG termini and relatively variable sequences at the 5′ss. However, the true significance of this finding did not become evident until a provocative hypothesis by Hall and Padgett,^2^ who suggested that such introns may be recognized by the factors that are specific to this type of introns. This hypothesis set the stage for the discovery that the genomes of most eukaryotes actually harbor two different types of introns, termed U2 and U12 type, that are removed by two separate spliceosomes.

The significance of having two parallel intron types and two machineries for their removal was initially perplexing, especially since the U12-type introns are present at very low frequencies. The function and significance of the U12-dependent spliceosome are still far from being fully understood, but there are several lines of evidence indicating that U12-type splicing has essential functions. These are related to the conservation of U12-type introns in distantly related organisms and their presence in specific types of genes, as well as to the slow kinetics of their removal, which may regulate the expression of the genes containing them. Here, we review the current knowledge on U12-type introns and the function of the U12-dependent spliceosome. In particular, we discuss the recent findings on the regulation of the U12-dependent splicing, its function in development and disease, and the impact of these findings on understanding the evolution of the two spliceosomes.

## U12-TYPE INTRON SEQUENCES: DEFINITION AND CHARACTERISTICS

**FIGURE 1:**
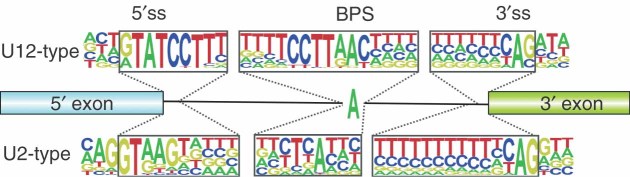
Consensus sequences of human U12- and U2-type introns. The height of the letters in each position indicates the relative frequency of individual nucleotides in that position. For frequency calculation the U12-type splice site sequences were obtained from U12DB,^7^ and the corresponding 5′ss and 3′ss frequencies for U2-type introns from the Splice Rack database (Ref 8; http://katahdin.mssm.edu/splice/index.cgi?database=spliceNew2) and the branch point sequences (BPS) data from Gao et al.,^9^ The sequence logos were generated using the enoLOGOS web server.^10^

Although the U12-type introns were first identified on the basis of terminal AT–AC dinucleotides (therefore initially referred to as AT–AC or atac introns) it was soon realized that these termini are not exclusively present in U12-type introns.^3^ Rather, the defining features of U12-type introns are the 5′ss and branch point sequences (BPS), which are more conserved than in most other introns^4^ (Figure 1). GT–AG were also found to function as the terminal dinucleotides, and are actually the more common subtype.^5^ Thus, a nomenclature was adopted in which the original spliceosomal introns are referred to as U2-type introns and the novel introns as U12-type introns. The introns are spliced by the U2- or U12-dependent spliceosomes, and are also often referred to as the ‘major’ and ‘minor’ spliceosomes, respectively.^5^,^6^

Naturally occurring U12-type introns mostly belong to either the AT–AC or GT–AG subtypes. A few natural examples of U12-type introns with other combinations of terminal residues have been reported, and non-canonical termini have also been shown to support U12-dependent splicing experimentally, although often with reduced efficiency.^11^–^15^ However, combinations of the main subtypes (i.e., AT–AG or GT–AC) appear to be disfavored. In addition to their distinct 5′ss and BPS, U12-type introns are also characterized by the lack of a distinct polypyrimidine tract (PPT) that is typically found upstream of U2-type 3′ splice sites. The distance from a U12-type BPS to the 3′ss has been shown to be an important factor for the recognition of U12-type introns, and is significantly shorter than in U2-type introns, with an optimum distance of 11–13 nt.^11^–^13^,^16^,^17^

## DISTRIBUTION OF U12-TYPE INTRONS

**FIGURE 2:**
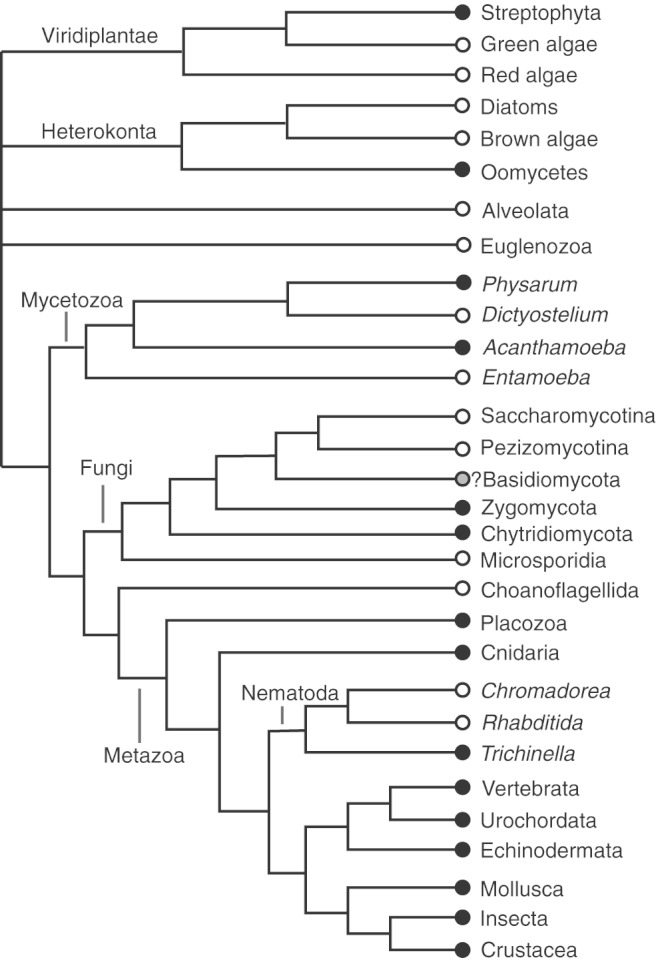
Phylogenetic distribution of U12-type introns and splicing factors, with special emphasis on the recurrent loss of U12-dependent splicing in the fungi-metazoa lineage. Filled circles in the schematic tree indicate taxa in which U12-type introns and/or splicing factors have been identified, while open circles indicate taxa in which they have not been observed. Names of individual genera have been given in italics. The lengths of the branches do not indicate true phylogenetic distances. The tree is based on the data obtained from Refs 19 and 23.

Currently, U12-type introns have been identified in all major eukaryotic taxa, including plants, fungi, and animals, as well as a few deep-branching single-celled eukaryotes, but are nonetheless absent in many species, including such common model organisms as *Caenorhabditis elegans* and *Saccharomyces* *cerevisiae*^4^,^8^,^13^,^16^,^18^,^19^ (Figure 2). The putative U12-type introns can readily be recognized computationally on the basis of the 5′ss and the BPS that are conserved among different organisms. An examination of the U12-type introns in the Splice Rack database (Ref 8; see Figure 1) revealed approximately 700–800 putative genes carrying U12-type introns in human and mouse, and ca 300 in *Arabidopsis thaliana,* while in *Drosophila* *melanogaster* only 19 U12-type introns have been described.^20^,^21^ However, these are estimates based on relatively stringent bioinformatic criteria, as there is little experimental evidence on the recognition of weak U12-type splice sites. Therefore, with less stringent criteria the number of putative U12-type introns could be significantly larger as suggested recently in *A.* *thaliana*.^22^

The genes containing U12-type introns are enriched in certain functional classes and pathways as described originally by Burge et al.^4^ They are mainly present in genes related to ‘information processing functions’, such as DNA replication and repair, transcription, RNA processing, and translation, but can also be found in genes related to cytoskeletal organization, vesicular transport, and voltage-gated ion channel activity. In contrast, U12-type introns are almost absent in genes related to basic energy metabolism and biosynthetic pathways.^4^,^24^,^25^ Typically only one U12-type intron is present in an individual gene. However, in humans, there are approximately 50 genes containing two U12-type introns and a few cases with three U12-type introns. This is most common in genes belonging to the voltage-gated ion channel superfamily.^24^,^25^ Together, these observations have led to the suggestion that U12-type introns may have a role in regulating the expression of specific sets of genes.

## SPLICEOSOME ASSEMBLY AND CATALYSIS

### The Major Spliceosome

**FIGURE 3:**
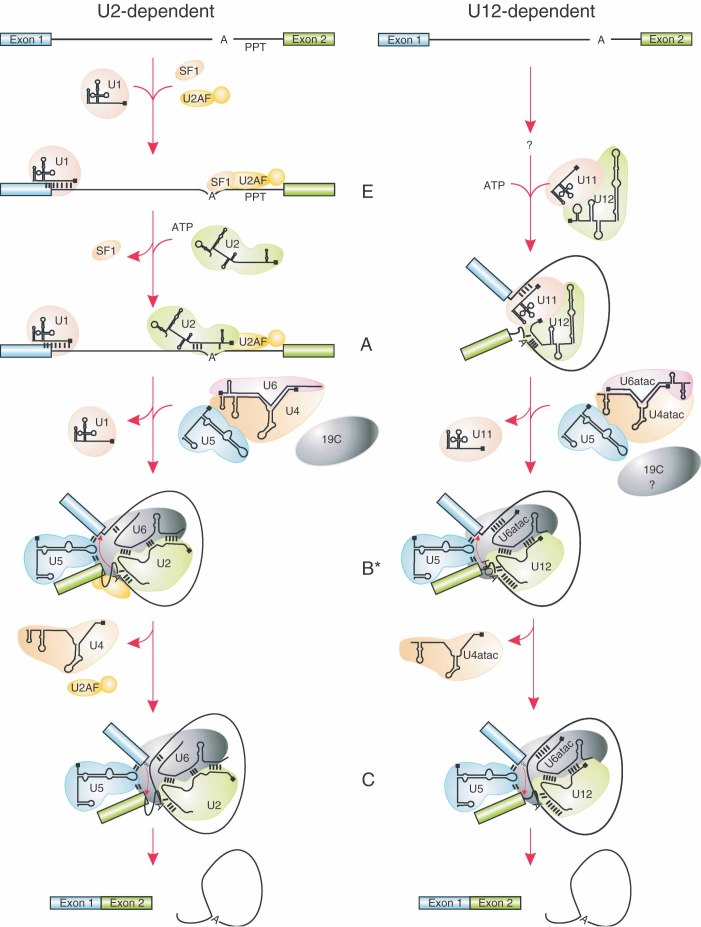
Spliceosome assembly. The interactions of the spliceosomal snRNPs and some selected non-snRNP protein complexes at various stages of spliceosome assembly (complexes E, A, B*, and C) are depicted schematically for both the U2- and U12-dependent spliceosomes. The Prp19/CDC5 complex is indicated by ‘19C’. Its association with the U12-dependent spliceosome is inferred from the major spliceosome and is therefore indicated with a question mark. (Adapted with permission from Ref 27. Copyright 2003 Macmillan Publishers Ltd)

Introns are recognized and excised by a large molecular machine called the spliceosome that is composed of five small nuclear ribonucleoproteins (snRNPs) and additional non-snRNP protein components.^26^ Each of the five snRNPs contains one small nuclear RNA (snRNA) and a number of protein components. The U2-type snRNPs are U1, U2, U4, U5, and U6, the latter three of which form a U4/U6.U5 tri-snRNP. The spliceosome is formed by the sequential interactions of the snRNAs and spliceosomal proteins with the pre-mRNA substrate and with one another. In U2-type introns, the 5′ss is initially recognized by the U1 snRNP, while the BPS, PPT, and 3′ss are recognized by the protein factors SF1, U2AF^65^ and U2AF^35^, respectively, together forming the spliceosomal commitment (or E) complex. During the formation of the pre-spliceosome, or A complex, U2 snRNP replaces SF1 at the BPS. At later stages, the U4/U6.U5 tri-snRNP stably associates with the spliceosome (B complex). Rearrangements in RNA and protein interactions lead to the formation of the catalytically active spliceosome (B* complex) that catalyzes the first transesterification reaction, followed by the second transesterification in the C complex (Figure 3).

### Recognition of U12-Type Introns by the Minor Spliceosome

**FIGURE 4:**
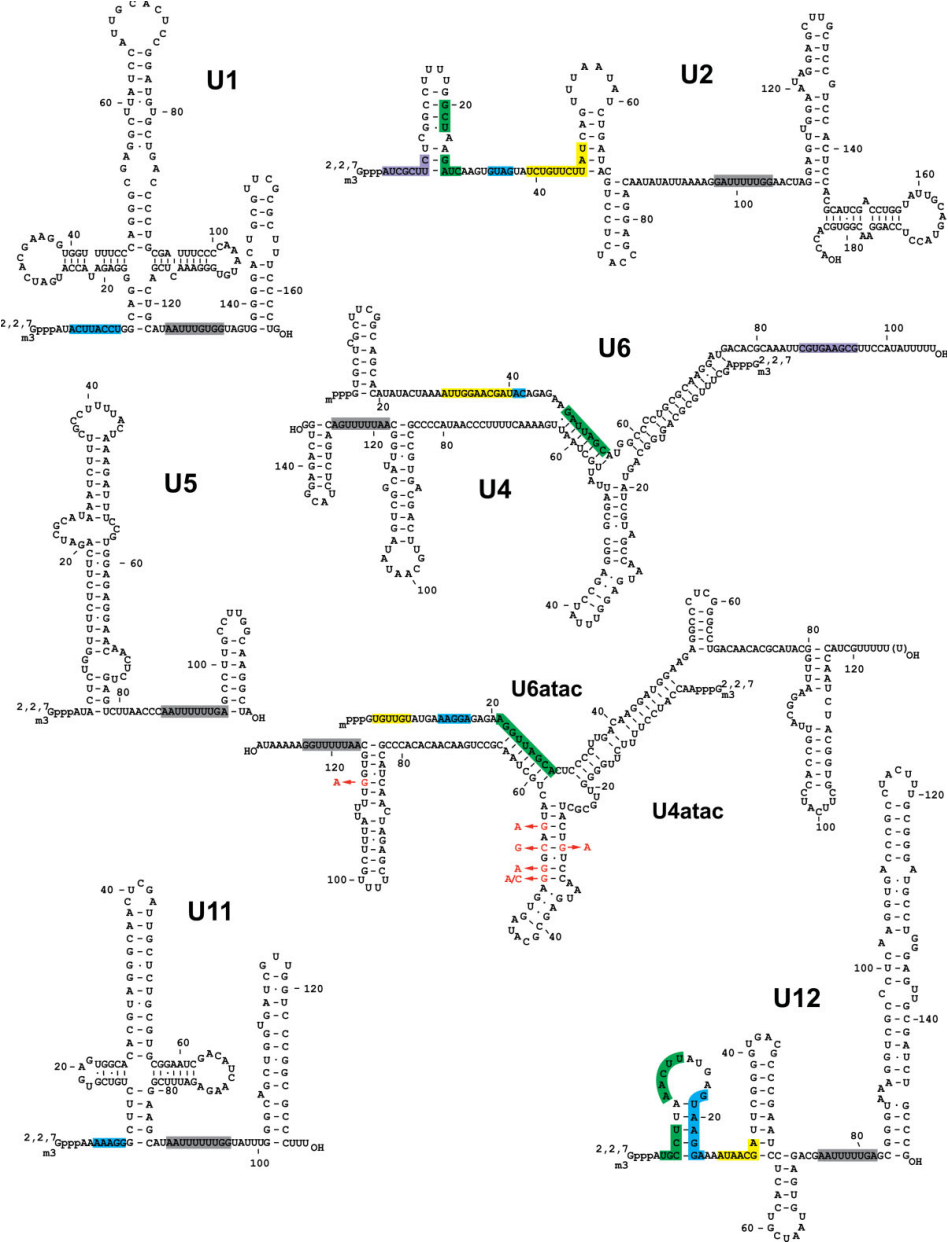
The predicted secondary structures of the human spliceosomal snRNAs. The binding sites for Sm proteins are shaded in gray, and the sequences interacting with the 5′ss or BPS in cyan. Sequences involved in various U2/U6 or U12/U6atac interactions are indicated by green (helix I), purple (helix II), and yellow shading (helix III), similar to Figure 5. Nucleotide modifications are omitted. (Structures are based on data originally published in Ref 34 for U1, U2, and U5, Ref 18 for U11, Ref 35 for U12, and Ref 29 for U4, U6, U4atac, and U6atac). The locations and identities of the Taybi-Linder syndrome or microcephalic osteodysplastic primordial dwarfism type I (TALS/MOPD1) mutations in the U4atac snRNA are from Ref 36 and are indicated in red.

The U12-dependent spliceosome contains four specific snRNPs, U11, U12, U4atac, and U6atac, each of which contains a specific snRNA component that is equivalent to but distinct from its U2-type counterpart, i.e., U1, U2, U4, and U6, respectively. U5 snRNP is shared between the two spliceosomes. Although the sequences of the snRNAs with equivalent function are quite divergent in the two spliceosomes, they share a common overall secondary structure (Figure 4). U4atac, U6atac, and U5 associate into a tri-snRNP, similar to the major U4/U6.U5, and the protein composition of the major and minor tri-snRNPs appears to be very similar, if not identical.^28^ Moreover, it has also been shown that equivalent stem-loop structures of U4 and U4atac function as binding platforms for proteins that are required for the tri-snRNP formation.^28^,^29^ In contrast, while the major U1 and U2 snRNPs are distinct snRNPS, their counterparts are present in the nucleus as a U11/U12 di-snRNP.^30^,^31^ Interestingly, only seven protein components have thus far been reported to be specific to the minor spliceosomes (Table 1), and they are all located in the U11/U12 di-snRNP.^32^,^33^ U11/U12 also lacks all the U1-specific proteins and some U2-associated proteins (Table 1), making it the most divergent component of the minor spliceosome, in comparison to its counterparts in the major spliceosome.

**FIGURE 5:**
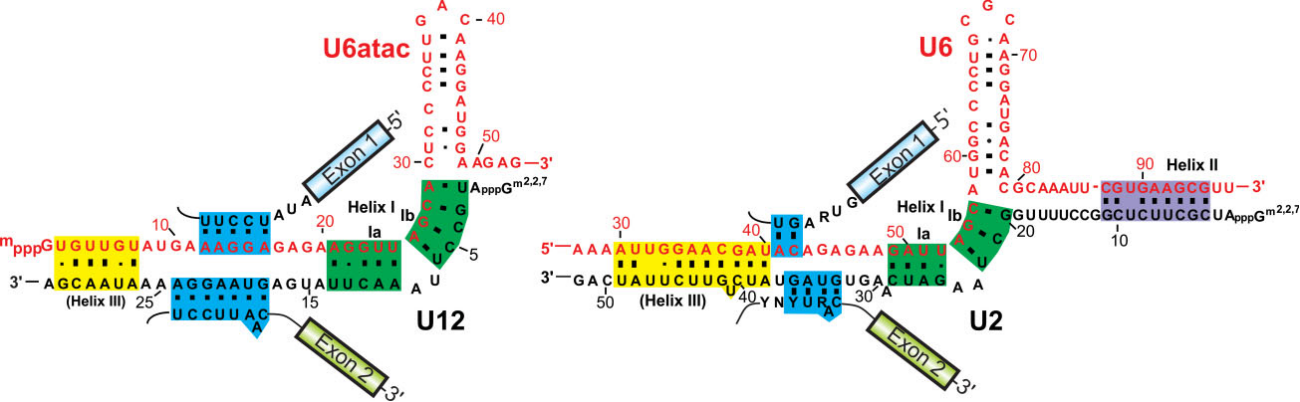
RNA–RNA interactions in the catalytic cores of the minor and major spliceosomes. Interactions between snRNAs and the 5′ss or BPS are indicated by cyan shading. U2/U6 or U12/U6atac interactions are indicated by green (helix I), purple (helix II), and yellow shading (helix III), as in Figure 4. The minor spliceosome structure is based on data published in Ref 59. The U12/U6atac helix III structure is controversial as it is not conserved in plants,^67^ but mutations in U12 snRNA that weaken this structure reduce the splicing activity in mammals.^35^

**1:** Proteins of the U1, U2, and U11/U12 snRNPs

12S U1	17S U2	18S U11/U12	Functions (with Selected References)
Sm proteins^1^	Sm proteins^1^	Sm proteins^1^	snRNP core components^26^
U1-A (SNRPA)			Structural; RNA-binding^37^
U1-C (SNRPC)			5′ss recognition^37^
U1-70K (SNRNP70)			Structural; SR protein interactions^37^,^38^
	U2A′ (SNRPA1)		Structural; RNA-binding
	U2B^′′^ (SNRPB2)		Structural; RNA-binding
	SF3a complex^2^		BPS binding^39^
	SF3b complex^2^	SF3b complex^2^	BPS binding^33^,^39^
		20K (ZMAT5)	Unknown; homology to U1C^32^
		25K (SNRNP25)	Unknown
		31K (ZCRB1)	Unknown; RNA-binding^40^
		35K (SNRNP35)	SR protein interactions, homology to U1-70K^33^,^41^,^42^
		48K (SNRNP48)	5′ss recognition^43^
		59K (PDCD7)	Structural, binds 48K and 65K^43^,^44^
		65K (RNPC3)	Structural, binds U12 snRNA^44^
		Urp^3^ (ZRSR2)	3′ss recognition^45^
	hPrp43^4^	hPrp43 (DHX15)	
		Y Box-1^3^ (YBX1)	

The Hugo names of proteins have been provided in parentheses.

1 Sm proteins B/B', D1, D2, D3, E, F, and G,

2 Multi-subunit complexes.

3 Also present in the major spliceosome, but not in U1 or U2 snRNPs.

4 Almost stoichiometric presence in the 17S U2. Other proteins associated with U2 in substoichiometric amounts are omitted.^46^

The overall assembly pathways of the two spliceosomes are similar, and the main difference is the absence of a separate commitment complex in the minor spliceosome. Instead, the preformed U11/U12 di-snRNP binds the intron as a unit, and the 5′ss and BPS are recognized in a cooperative manner within the A complex, although U11/5′ss basepairing still precedes the formation of stable U12/BPS basepairing.^47^ The initial basepairing interactions at the 5′ss are also different in the two spliceosomes: in contrast to the U1, the U11 snRNA does not basepair across the exon– intron boundary or even with the first three nucleotides of the intron.^2^,^48^ Instead, these nucleotides are recognized specifically by the U11-48K protein, which likely also stabilizes the U11/5′ss helix.^43^,^49^ Surprisingly, even though U11-48K does not share significant sequence homology with the U1-specific protein U1-C, both appear to stabilize the binding of their respective snRNAs in a similar manner through their zinc finger domains.^37^,^49^

Interaction of the U12 snRNA with the BPS is similar to the U2/BPS basepairing in the major spliceosome, resulting in the exclusion of the branch point (BP) adenosine from the U12/BPS helix.^50^,^51^ There seems to be flexibility in the choice of the BP adenosine, as either one of the two A residues present in the consensus BPS can be used as a BP, depending on the intron in question.^52^ BPS recognition is likely stabilized by proteins of the SF3b complex, which is present in both spliceosomes (Table 1), and is known to bind to the BPS in the major spliceosome, with the protein factor p14 shielding the BP adenosine from premature activation.^39^,^53^,^54^ U12-type introns do not have PPTs, and U2AF is not required for the recognition of U12-type introns.^55^ However, a U11/U12 di-snRNP component related to U2AF^35^, Urp,^32^ is required for A complex formation and 3′ss recognition.^45^ Urp is not specific to the minor spliceosome, but its function is different than in the major spliceosome, where it apparently displaces U2AF from the 3′ss after the first catalytic step.^45^

In the major spliceosome the U1 and U2 snRNPs recognize the 5′ss and BPS independently, and non-snRNP proteins are required for their association to each other.^56^ In contrast, the U12-type 5′ss and BPS are linked through the internal components of the U11/U12 di-snRNP already at the earliest phase of intron recognition. The protein factors U11-59K and U11/U12-65K interact at the interface of the two snRNPs.^44^ U11-59K further interacts with the U11-48K protein,^43^ while U11/U12-65K directly binds to the 3′ terminal stem loop of U12 snRNA.^44^ Owing to the compact structure of the di-snRNP,^57^ further protein and/or RNA interactions are likely to contribute to the association of the two snRNPs. Furthermore, the binding sites for the 5′ss and BPS must be close to each other within the U11/U12 structure, as the very 5′ end of U12 snRNA can be cross-linked to the pre-mRNA 2 nt upstream of the 5′ss already in the A complex, suggesting that the BPS and 5′ss must be within 50 Å of one another.^58^ Thus, the 5′ end of U12 is already close to the position required for the formation of the catalytic core of the spliceosome.

### Assembly of the Catalytic Core is Similar in the Two Spliceosomes

The formation of the catalytically active spliceosome is thought to follow a pathway similar to that of the major spliceosome. After initial recognition of the splice sites by the U11/U12 di-snRNP, the U4atac/U6atac.U5 tri-snRNP associates with pre-spliceosome to form complex B^59^ (Figure 3). A terminal stem-loop structure in the U6atac snRNA (possibly together with U4atac snRNA sequences) contains a signal that directs the U4atac/U6atac.U5 tri-snRNP to the minor and not to the major pre-spliceosome.^60^ A large number of structural rearrangements convert the pre-catalytic spliceosome (complex B) to the catalytic configuration (complexes B* and C) in a manner similar to the major spliceosome. U6atac snRNA replaces U11 at the 5′ss, U4atac/U6atac structure is unwound, and U12 and U6atac basepair with each other to form the ‘catalytic core’ structure in which the reactive A residue at the BP and the 5′ss are juxtaposed for the first step of the catalysis.^59^,^61^–^63^ During this process both the U11 and U4atac snRNAs are released from the spliceosome. In the major spliceosome, specific helicases drive forward spliceosome assembly and the transesterification reactions.^64^ There is virtually no data showing whether helicases have similar activities in the minor spliceosome. However, given that most proteins are shared^28^,^32^ and no helicases specific to the minor spliceosome have been identified, it is likely that the helicase activities are also the same during the assembly of the two spliceosomes.

Although the formation of the catalytic core appears to be somewhat more flexible in the U12-dependent spliceosome,^61^ the structure and function of the core are likely to be highly similar. Indeed, both spliceosomes employ the same two-step transesterification mechanism for intron removal,^51^ and U12-dependent splicing is even supported by the modified U6atac snRNAs in which the functional domain has been replaced by that of U6 snRNA.^65^ The U12/U6atac interactions in the catalytic core of the U12-dependent spliceosome resemble those in the major spliceosome, although the helix II structure present in the U2/U6 complex cannot be formed in the minor spliceosome (Ref 59; Figure 5). Nevertheless, the similarity of snRNA structural domains suggests that, like the major spliceosome,^66^ the minor spliceosome is likely to use RNA-based catalysis.

### Removal of U12-Type Introns Is Slow

Both intron types are spliced co-transcriptionally within the nucleus (Ref 68 and Box 1) and might be expected to display similar kinetics. However, the removal of U12-type introns appears to be significantly slower. Early *in vitro* splicing experiments documented a splicing rate for U12-type introns that was three- to fivefold slower than that of U2-type introns.^47^,^51^,^69^ Similar observations were also made in *in vivo* experiments, where approximately twofold higher levels of unspliced U12-type introns were detected in the steady-state transcript pools isolated from insect and mammalian cells.^69^–^71^ This is consistent with the observation that co-transcriptional splicing of U12-type introns is at least twofold slower than that of U2-type introns.^68^ Thus far, the reason for the lower efficiency remains unknown. Minor spliceosome snRNPs are ca 100-fold less abundant than the major snRNPs,^31^,^59^ and this could underlie the observed kinetic differences. However, the observation that a further 10-fold reduction in the levels of U4atac snRNA has no apparent effect on the efficiency of endogenous U12-dependent splicing argues against this simplistic explanation.^70^ The slower rate could also be related to the kinetic effects caused by the less flexible recognition phase of U12-type introns or by the inability to form some of the structures present in the catalytic core of the major spliceosome. Regardless of the underlying mechanism, Patel et al.^71^ have suggested that the slower rate of splicing could constitute a rate-limiting mechanism for the expression of genes containing U12-type introns. In this model, the transcripts containing unspliced U12-type introns would get trapped in the nucleus where they could be targeted by nuclear surveillance mechanisms.^72^

**BOX 1**

**NUCLEAR LOCALIZATION OF THE U12-DEPENDENT SPLICEOSOME**

A provocative hypothesis by König et al. suggested that the U12-dependent spliceosome is located in the cytoplasm and that the splicing of U12-type introns also takes place there.^73^ This suggestion was based on *in situ* hybridization studies, mostly with zebrafish, as well as cell fractionation and reverse transcription polymerase chain reaction (RT-PCR) analyses. The publication contradicted many of the earlier studies on minor spliceosome component localization and function (See Ref 74 and references therein) and spurred a lively debate on the localization of the U12-dependent spliceosome. Subsequent publications failed to reproduce the key findings of the paper and instead demonstrated nuclear localization for U12-type spliceosome snRNP and protein components in mammalian cells and tissues,^75^ nuclear splicing of U12-type introns in *Xenopus* oocytes,^76^ and co-transcriptional splicing of U12-type introns.^68^ Together, these subsequent studies provide firm evidence for the nuclear localization of the splicing of U12-type introns.

## EXON DEFINITION INTERACTIONS AND ALTERNATIVE SPLICING

Splicing enhancer and silencers sequences are important for defining splice sites both in constitutive and alternative splicing, and typically function by recruiting regulatory splicing factors, such as SR and hnRNP proteins. U12-type introns are not an exception in this regard, as U12-dependent splicing also responds to SR and hnRNP proteins that bind to splicing regulatory elements near the U12-type splice sites.^17^,^77^–^79^ A prototypical case of SR protein-mediated enhancement in the major spliceosome is the interaction of SRSF1 with the U1-70K protein, which enhances 5′ss recognition.^38^ The U11/U12 di-snRNP protein U11-35K is the putative paralog of U1-70K,^33^ and may have a similar function in stabilizing U11/5′ss binding. This notion is indirectly supported by the findings that U11-35K interacts with SR proteins in plants, including SRSF1 homologs,^41^,^42^ and that U12-type A complex formation is enhanced by SR proteins.^77^ As in the major spliceosome, SR proteins likely enhance intron recognition in multiple ways and through several spliceosomal factors, and have also been shown to directly contact U12-type 5′ss and BPS.^55^,^77^

**FIGURE 6:**
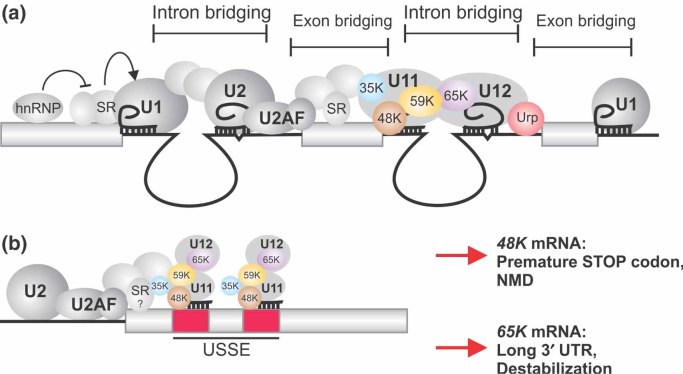
Exon definition interactions and regulation of U12-type factors by alternative splicing nonsense-mediated decay (AS-NMD). (a) Exon definition interactions form between U2- and U12-dependent spliceosomes in both the upstream and downstream direction, aided by SR protein interactions.^77^,^80^ Intron bridging differs in U12-dependent splicing as a consequence of the cooperative recognition by the U11/U12 di-snRNP.^47^ (b) The di-snRNP activates alternative splicing by binding to the U11 snRNP-binding splicing enhancer (USSE) element in the U11-48K and U11/U12-65K transcripts and recruits U2-type splicing factors to the upstream 3′ss. Alternatively spliced products are degraded.^81^

A particular problem related to the processing of nascent transcripts is that introns (especially those of vertebrates) can be extremely long, such that it can take an hour for an entire intron to be transcribed. Therefore, initial splice site definition typically takes place over the considerably shorter exons, with 3′ss-recognizing factors in the upstream intron interacting with the 5′ss-recognizing factors in the downstream intron (Figure 6(a)). Again, U12-dependent splicing has been shown to be enhanced by the presence of flanking U2-type introns,^17^,^80^ an effect which is likely mediated by the SR proteins or other auxiliary factors. The most detailed evidence for exon definition interactions between components of the two spliceosomes comes from the alternative splicing (AS) of the transcripts coding for the minor spliceosome components U11-48K and U11/U12-65K.^81^ In this system, an alternative U2-type 3′ss is activated by the U11/U12 di-snRNP bound at an atypical downstream splicing enhancer element consisting of a tandem repeat of U12-type 5′ss, designated as U11 snRNP-binding splicing enhancer (USSE; Figure 6(b)). The alternatively spliced transcripts are degraded by the nonsense-mediated decay (NMD) pathway or other RNA degradation machineries, and this system thus functions as a regulatory feedback mechanism for minor spliceosome components.

Given that AS is extremely prevalent in humans, and the extensive interactions of the minor spliceosomes with other splicing factors, it is somewhat surprising that AS events involving U12-type introns appear to be almost entirely absent. This may be because of the more rigid sequence and distance constraints on the U12-type 5′ss, BPS, and 3′ss. AS events involving the use of an alternative 3′ss have been observed, but it is not entirely clear whether they are the result of true regulated AS or of splicing errors, especially since the selection of the U12-type 3′ss has been shown to be prone to errors.^13^,^16^,^82^,^83^ Exon skipping, which is the most common type of AS observed with major introns, has been reported only in a few instances involving U12-type introns, and the functional significance of these AS events is not known.^14^,^16^ The absence of exon skipping is likely to be due to the general incompatibility of U12 and U2-type splice sites and the scarcity of genes containing more than one U12-type intron. Alternative usage of mutually exclusive U12-type and U2-type splice sites has been observed in at least three cases. In vertebrates, the members of the *JNK* gene family contain a hybrid intron with a U12-type 5′ss and U2-type BPS and 3′ss. These can be spliced in a mutually exclusive fashion to either the 3′ss of the downstream U12-type intron or to the 5′ss of the upstream U2-type intron, respectively,^82^ and at least in mice these isoforms show tissue specificity, with the former isoform preferentially expressed in neurons. The *D. melanogaster* genes *prospero* and *dUrp* (which encodes the Urp protein present in both spliceosomes) contain overlapping U12 and U2-type introns or ‘twintrons’.^2^,^14^,^84^ The U12-dependent *dUrp* mRNA isoform is apparently destroyed by NMD,^14^ while *prospero* alternative splicing results in distinct developmentally regulated protein isoforms with amino-acid differences in the homeodomain region.^85^,^86^

## PHYSIOLOGICAL SIGNIFICANCE OF THE MINOR SPLICEOSOME

### Minor Splicing Is Essential for Development

Knockdown of protein components specific to the minor spliceosome leads to reduced proliferation of cultured cells,^32^,^43^ and thus minor splicing is an essential process. Proper U12-dependent splicing is also required for development, as shown in humans^87^,^88^ and in *D. melanogaster*,^84^ despite the fact that the latter only contains a handful of U12-type introns.^20^,^21^ Blocking minor splicing in zebrafish embryos resulted in developmental defects at a distinct stage, in comparison to blocking the major spliceosome.^73^ Knockdown of the U11-31K protein in *A. thaliana* also led to abnormal growth of the plant, but only after the bolting stage.^40^ These findings indicate that minor splicing has specific functions in regulating developmental processes. These functions may be mediated in part by alternative splicing, as with the *prospero* transcripts in *D. melanogaster*. However, it is likely that another, even more significant factor affecting developmental processes is the slower kinetics of minor splicing, which can limit not only the expression of the genes containing them^68^,^70^,^71^ but also downstream pathways. Indeed, defects in minor splicing have been shown to perturb the expression of multiple metabolic genes (which themselves do not contain U12-type introns) in *D. melanogaster*, likely underlying the developmental arrest.^84^,^89^ The developmental defect following the U11-31K knockdown in *A. thaliana* was also associated with downregulation of a specific set of genes related to gibberellic acid metabolism.^40^ Thus, U12-dependent splicing is necessary for multiple processes required for the viability and development of multicellular organisms, and can also affect specific cellular pathways.

### Minor Splicing and Human Disease

From a medical point of view, it would be interesting to know to what extent the activity of the minor spliceosome contributes to regulating the expression of genes in different human tissues. This question remains mostly unanswered. Some protein components specific to the minor spliceosome (31K and 65K) have been observed to be differentially expressed in different tissues,^90^,^91^ and U12-type intron-containing genes in bone marrow CD34 positive cells and B lymphoblasts,^24^ but the correlation between minor splicing and gene expression in different tissues has not been analyzed systematically. To date, only a few diseases have been linked to defects in minor splicing, some of them displaying tissue-specific symptoms, while others have a wide range of system-wide defects. Mutations in U12-type 5′ss in the *LKB1* and *SEDL* genes cause Peutz-Jeghers syndrome and spondyloepiphyseal dysplasia tarda, respectively,^83^,^92^,^93^ and these diseases likely arise from the inactivity or insufficiency of the respective gene products.

A more complex class of diseases are those arising from defects in minor spliceosome components of which the TALS/MOPD1 (Taybi-Linder syndrome or microcephalic osteodysplastic primordial dwarfism type I) is a recent example. This severe developmental disorder is caused by recessive mutations in the U4atac gene (Refs 87 and 88; see Figure 4). Unlike their counterparts in the major spliceosome, each minor snRNA is expressed from a single locus in the genome,^88^,^94^,^95^ and the mutations in such loci can therefore potentially disable the function of the snRNA in question. In the case of TALS/MOPD1, the mutations are mostly in the 5′ stem loop of U4atac (shown in Figure 4) and are predicted to prevent the binding of tri-snRNP-specific 15.5K and 61K proteins which could lead to a defect in the formation of U4atac/U6atac.U5 tri-snRNP,^36^,^96^ but experimental evidence for this is still lacking. This presumed defect in snRNP assembly decreases the activity of the U12-dependent spliceosome almost 10-fold,^88^ but still allows correct splicing of U12-type introns in most genes, albeit at significantly reduced levels. This resembles a situation with *D. melanogaster* carrying mutations in U6atac snRNA.^70^ An open question is whether the developmental defects observed in this disorder are due to a large-scale reduction in the expression of U12-type intron-containing genes or just a few affected genes in each tissue as has been suggested for *D. melanogaster*.^89^ A comparable case is spinal muscular atrophy (SMA), which arises from the mutation of the gene coding for the general snRNP assembly factor SMN, and is characterized by multiple defects in mRNA processing, including widespread changes in alternative splicing,^97^,^98^ but initially only affects spinal cord neurons. SMA has been reported to involve a decrease in the levels of the minor tri-snRNP^99^ and possibly U11/U12 di-snRNP levels as well,^98^,^100^ together with defective splicing of a few, but not all, U12-type introns.^99^ While the direct link of these findings on the pathogeny of SMA is not entirely clear, it is possible that the defect in the splicing of some U12-type introns in neurons either causes or contributes to the disease phenotype.

## EVOLUTION OF THE MINOR SPLICEOSOME

It is clear that the U12-dependent spliceosome is crucial for the viability and development of many multicellular organisms. However, intriguing questions about the minor spliceosome remain: Where did it originate? Why is it not present in all eukaryotes, and do its function and importance vary in different evolutionary lineages? We do not currently have clear answers to these questions, but recent advances in large-scale sequencing and bioinformatics are paving the way toward deeper understanding of the significance of the minor spliceosome.

### Origins and Conservation of the Minor Spliceosome

The two spliceosomes have been proposed to originate from group II self-splicing introns, but it is not clear whether they arose simultaneously or sequentially, and whether this took place in one ancestral eukaryote or separate lineages that later fused.^4^,^101^–^103^ However, based on the distribution of introns and spliceosome components, it is evident that both U2- and U12-type introns and spliceosomes were present in the last common eukaryotic ancestor (Refs 18, 19, and 23; Figure 2). U12-type introns show remarkable conservation over long evolutionary distances: the rate of intron loss or conversion has been very low in all studied vertebrates, and while *D. melanogaster* has only 19 U12-type introns, almost all of them have orthologous U12-type introns in human.^14^ Most remarkably, 20 U12-type introns are conserved in orthologous positions between human and *A. thaliana*.^16^,^104^ Also, although the sequences of U12-type snRNAs are more diverged in distantly related organisms than their U2-type counterparts, their structures are highly conserved.^18^,^20^,^41^,^67^

### Repeated Loss of U12-Type Introns

Despite the high overall conservation of U12-type introns, they are entirely absent from many eukaryotes whose relatives nonetheless have them, suggesting repeated loss of U12-dependent splicing during evolution. The loss seems to have been most prevalent among diverse eukaryotic microbes, but some animals, including some but not all nematodes, have also lost their U12-type introns (Refs 18 and 19; Figure 2). The loss of U12-type introns has also been extensive in *Diptera*, which typically only have 15–20 U12-type introns in total in their genomes, several fold less than in most other insects.^14^,^21^ The precise reasons behind these losses remain enigmatic. However, it has been suggested that introns confer a slight deleterious effect for the fitness of the organism,^101^ and could thus be removed more efficiently in organisms with large effective population sizes, such as microbes and small invertebrates.^14^ Presumably, U12-type introns may be even slightly more deleterious, due to their slower excision and lower accuracy. In support of this, the loss of U12-type introns generally correlates with reduction in overall intron frequency, but U12-type introns still tend to be lost to a larger extent.^14^,^21^

In most cases, the mechanism of U12-type intron loss involves a simple deletion of the intronic sequence, apparently through the homologous recombination of reverse transcribed mRNAs.^14^ Another pathway is the step-wise conversion of U12-type introns into U2-type introns. Natural examples as well as experimental approaches have also shown that simple point mutations in the 5′ss of a U12-type intron, especially in the GT–AG subtype, suffice to turn it into a U2-type 5′ss,^4^,^5^,^14^ as U2-type introns have much more degenerate splicing signals. Subtype switches from a U12-type AT–AC intron to U12-type GT–AG are also observed.^4^,^8^,^14^ However, such changes appear to be rare, possibly due to the poor splicing efficiency of the intermediate forms (e.g., AT–AG).^11^,^17^,^21^,^105^ Sometimes intron conversion can also occur by the activation of a cryptic U2-type 5′ss near the original U12-type 5′ss.^16^,^19^ Given that the chance of creating the highly stringent U12-type 5′ss from a U2-type 5′ss is low, conversion in the reverse direction is a highly unlikely event. In fact, only one instance of a novel U12-type intron has been observed (in the *dUrp* twintron).^14^ Thus, without natural selection acting in favor of maintaining U12-type introns, they are likely to be converted into U2-type introns during evolution.

### Conservation of Regulatory Features

Many splicing factors are regulated through a feedback mechanism, in which they bind to regulatory elements within their own pre-mRNA, resulting in alternatively spliced, unstable mRNA isoforms. Interestingly, all canonical SR and hnRNP proteins, as well as a number of core spliceosomal factors, are regulated in this manner, and in many cases the regulatory elements are also evolutionarily conserved.^24^,^106^–^110^ While most of the splicing factors thus regulated are common to both spliceosomes, certain spliceosome-specific proteins have also been shown to be alternatively spliced to produce unstable mRNA isoforms. These include, e.g., U1-70K and U11/U12-25K, for which the AS events are also evolutionarily conserved between human and mouse.^108^ The conservation of the AS events suggests that they have an important function in regulating the levels of these proteins, and may consequently affect the activity of the specific spliceosome in question. The feedback mechanism regulating the levels of minor spliceosome-specific proteins U11-48K and U11/U12-65K through the USSE element (Figure 6(b)) is conserved over extremely long evolutionary distances, in plants as well as in animals.^81^ Interestingly, the USSE is most conserved in vertebrates,^81^ which also show the most extensive conservation of U12-type introns,^8^,^14^,^105^ and is absent in *Diptera*, where both the overall numbers and interspecies conservation of U12-type introns are low.^21^ It is thus possible that this mode of regulation is important for the rate-limiting function of the minor spliceosome, without which the presence of U12-type introns would be mainly deleterious. Interestingly, the emergence of an AS-NMD-targeting U12-type intron in the *Drosophila dUrp*^14^ suggests that it, too, may function as a feedback mechanism for the minor spliceosome. Thus, it is possible that mechanisms limiting the activity of the minor spliceosome are essential for its function in regulating the expression of U12-type intron-containing genes.

## CONCLUSION

Historically, insight into the function and significance of the U12-dependent spliceosome has come primarily from the biochemical characterization of splicing factors and spliceosome assembly, and has recently been complemented by the increasing number of bioinformatic analyses. However, our understanding of the significance of minor splicing is far from complete in both regards. The function of many of the U12-specific components is still unknown, and more components might still remain undetected. Importantly, although there are many independent lines of evidence indicating that U12-type introns are important for the regulation of gene expression, definitive large-scale evidence on the connection between the activity of the minor spliceosome and gene regulation is still lacking. Are there more regulatory elements that respond to U12-type factors? In what other ways is the minor spliceosome regulated, and how does this correlate with the expression of U12-type intron-containing genes in different tissues? Could there be U12-type introns with more degenerate splice sites, and could they be involved in more extensive alternative splicing? An exciting way of studying these questions is now offered by the rapidly developing high-throughput sequencing and cross-linking methods, and will hopefully deepen our understanding on the significance of minor splicing in the coming years.
